# Development of a System for Storing and Executing Bio-Signal Analysis Algorithms Developed in Different Languages

**DOI:** 10.3390/healthcare9081016

**Published:** 2021-08-07

**Authors:** Moon-Il Joo, Satyabrata Aich, Hee-Cheol Kim

**Affiliations:** 1 Institute of Digital Anti-Aging Healthcare, Inje University, Gimhae-si 50834, Korea; joomi@inje.ac.kr; 2 Department of Computer Engineering, Inje University, Gimhae-si 50834, Korea; satybrataaich@gmail.com

**Keywords:** data mining, bio-signal analysis, bio-signal repository, execution engine, bio-signal monitoring

## Abstract

With the development of mobile and wearable devices with biosensors, various healthcare services in our life have been recently introduced. A significant issue that arises supports the smart interface among bio-signals developed by different vendors and different languages. Despite its importance for convenient and effective development, however, it has been nearly unexplored. This paper focuses on the smart interface format among bio-signal data processing and mining algorithms implemented by different languages. We designed and implemented an advanced software structure where analysis algorithms implemented by different languages and tools would seem to work in one common environment, overcoming different developing language barriers. By presenting our design in this paper, we hope there will be much more chances for higher service-oriented developments utilizing bio-signals in the future.

## 1. Introduction

With the incoming of the fourth industrial revolution, the technological development of the Internet of Things (IoT) and smart infrastructure makes us pay more attention to the technology in order to collect and analyze a huge amount of information [1,2]. Particularly in medical and healthcare fields, there has been a paradigm shift from cure-oriented to prevention-oriented medical practices, partly due to the emergence of wearable devices that can measure and acquire vital signs wherever and whenever the users are [3,4,5,6]. Wearables are now part of every individual since these devices provide more concrete analytics decisions about the individuals using the individual data, which could help in better decision making connection with the bio-signals [7]. Naturally, it enables better and high-quality medical and healthcare services utilizing the vital signs acquired.

Recently, such data have been accumulated exponentially with the help of the devices [8]. It is not that difficult to imagine the potential knowledge and information inferred by the analysis of the big data for disease prevention, health management, diagnosis, therapies, etc. [9,10,11,12]. Artificial intelligence has created a lot of positive impacts in clinical decision making, diagnosis, predictive medicine, etc., which is a good sign for developing personalized systems [13].

Personalized and customized healthcare services are expected to be common sooner or later. Since smartphones and wearable healthcare devices are already employed, the technology for collection and analysis of health information gathering is easier and advanced enough [14,15]. This situation promotes more research on various healthcare services utilizing and analyzing vital signs [16,17]. Global IT giants including Apple, Google, and Samsung are carrying out huge projects where healthcare platforms and services as well as wearable devices with biosensors are designed and developed [18,19].

The development of a bio-signal analysis algorithm is of prime importance to provide a seamless healthcare service. All bio-signal data have no meaning on their own. For example, electrocardiogram (ECG) signals and pulse wave data are time-series data, and health status cannot be analyzed using data alone. In this case, to analyze the health status, it is necessary to extract feature values by applying an analysis algorithm suitable for the data, since these services can be provided to the healthcare system using these characteristic values. Moreover, due to the fact that the development of bio-signal analysis algorithms is performed in multiple languages such as MATLAB and R, the source code conversion technology is a requirement to make the system development independent of the programming language. However, source code conversion techniques are primarily a very complex and redundant process and also depend heavily on the development tool which is used for deployment. Furthermore, due to the complexity, the management of the source codes of algorithms and the reusability of the source codes becomes very difficult. Therefore, to overcome the complex manual conversion process, this work implemented an algorithm specification for developing bio-signal analysis algorithms in different languages and a common execution engine that will be able to execute the algorithms written in different languages. The proposed architecture provides software architecture, by which one can reuse the bio-signal analysis algorithms developed by other developers in different languages such as MATLAB and R, without building a transformation process between multiple development environments.

The primary objective of the study is to develop a smart interface to run bio-signal analysis algorithms developed in different languages. An execution engine is developed to apply the smart interface. The execution engine can easily apply the bio-signal analysis technology developed in various algorithm development languages to the system using the source code conversion technology. This technology is expected to increase the reusability of analysis algorithms and the efficiency of system development.

When such a smart interface is provided, healthcare system developers can have more room to go further to higher service-oriented development using bio-signals. In addition, it is judged that the execution engine proposed in this paper can be used in various fields that require signal processing other than the healthcare field.

The remainder of this paper is organized as follows. First, we present the related works and backgrounds. Second, we describe the design of bio-signal storage where bio-signals with big sizes are stored and managed. Third, we discuss an architecture to support a smart interface among heterogeneous mining algorithms implemented in different languages. Fourth, the results and discussion of this paper are presented. Finally, we conclude with a description of the results.

## 2. Related Works

The conversion technique of the analysis of the vital sign algorithm source code is the task of changing the algorithm source code to match the system that provides healthcare services. Typically, the algorithms developed by MATLAB and Python are converted to C/C++ and applied to the system [20,21]. However, applying the source code converted to C/C++ to the system requires additional work by the developer to accommodate the system environment. We developed a system that can execute the source code of algorithms from multiple programming languages in the Java environment. The interface developed in the work is a Java-based interface, that can execute various bio-signal analysis algorithms from a single service definition. The work proposed in the paper allows the execution of bio-signal analysis source codes developed in the MATLAB and R programming using Java-based libraries running on Java Runtime Environment.

R programming was conducted to secure the interoperability between Java and R programming using rJava [22]. An interoperability study using rJava uses Java’s graphical user interface (GUI) to overcome the delicate graphic task, which is a disadvantage of R programming. This is a graphical representation of the data analyzed using JavaFX by R programming [23]. The study also analyzes the disease data of patients using R programming and shows the analyzed data using the GUI in Java [24]. 

The MATLAB control library can connect to the MATLAB engine in Java and execute the MATLAB source code [25]. A typical remote connection to a server installed with MATLAB was made to execute the MATLAB command using Java and MATLAB control [26].

The technology of executing the algorithm source code itself has been studied to perform more sophisticated graphical tasks or to use development tools remotely. As such, most studies have been conducted by choosing the development tool for the system environment being developed. So far, research into applying development tools developed in different languages is insufficient. Therefore, research needs to be done by applying the source code developed with various development tools to the system and executing the desired algorithms. This study will be the basic research to apply various algorithm managements to the system. 

**Figure 1:**
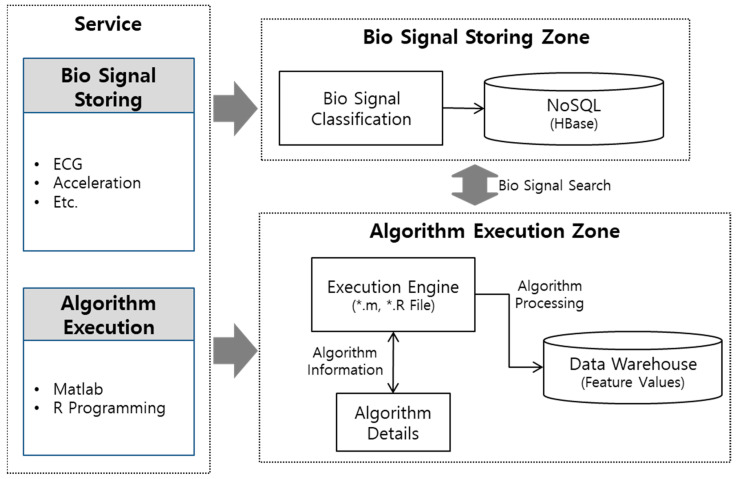
Architecture for analyzing bio-signal data by MATLAB and R programming.

This paper proposes an architecture for executing the source code itself, which is developed by MATLAB and R programming in the system, as shown in Figure 1. The proposed architecture is divided into an execution engine that executes the vital signs algorithm and a repository that stores the bio-signals.

## 3. Materials and Methods

The bio-signal analysis system proposed in this paper has two services, as shown in Figure 1. First, the bio-signal storage service collects bio-signals and stores the collected bio-signals in big bata-based NoSQL. Second, the algorithm execution service develops an execution engine for executing the bio-signal analysis algorithms developed in various development languages.

### 3.1. Bio-Signal Storage Design

The data accumulated by wearable health devices typically form big data. For instance, when the ECG sampling rate is 500 Hz, the system collects 500 pieces of data per second from an individual. Suppose it can gather them for a day. Then, the amount is 43,200,000 pieces of data. If it gathers them from more than 1000 people for a year or so, the amount of ECG data increases exponentially. The database storing such big data must secure scalability. As we know it, however, the relational database management system (RDBMS) has difficult aspects of processing such explosive vital sign data. Since RDBMS stores structured data, it uses data consistency and normalization and provides high performance. However, we face a huge problem to process unstructured data and big data beyond zettabytes [27]. There is a need for a new way of storing and processing big unstructured or semi-structured data, which we call NoSQL. NoSQL is a non-relational database where its table schema is not fixed, join operation is not supported, and its horizontal expansion is easy. Therefore, NoSQL is more suitable for processing a vast number of data [28].

NoSQL can be divided into three ways of storing: Key-value store, document store, and column store. The key-value store database stores, retrieves, and manages data as a key/value pair. A document store NoSQL database retrieves data by more complex conditions than key/value types, and its typical examples are MongoDB and CouchDB. Column store databases have a more powerful scalability in, for example, Cassandra and HBase.

**Figure 2:**
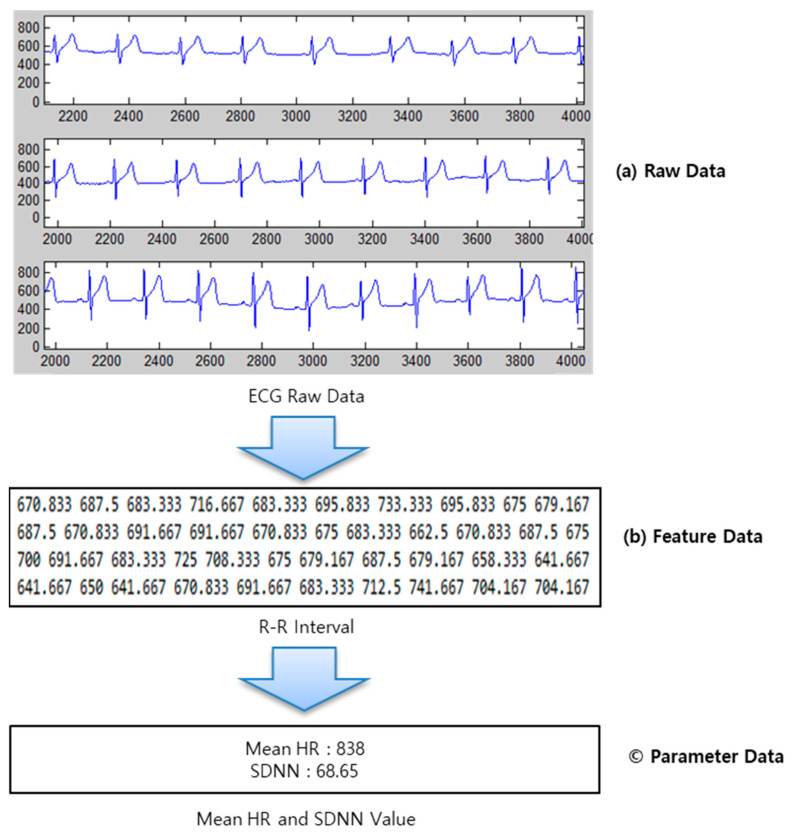
Characteristics of data according to the ECG signal analysis.

In this paper, according to the bio-signal characteristics of Figure 2, the bio-signal raw data and feature data are stored in a storage. Bio-signal raw data is stored in NoSQL based on big data, and feature data is stored in Datawarehouse for big data analysis. Since recent bio-signals reflect various bio-signals such as electrocardiogram, respiration, respiration, SpO2, etc. to analyze disease or health conditions. To facilitate the search and analysis of bio-signals, raw data and feature data should be stored separately. In addition, this bio-signal classification method is easy to further expand feature data according to various bio-signal analysis techniques.

#### 3.1.1. Design of Bio-Signal Raw Data Storage

In this paper, we designed a wide column database based on big data to store raw bio-signal data. This is due to the fact that the wide column database has excellent compression, distributed processing, aggregation processing (sum, count, avg, etc.), and query operation speed and scalability of large amounts of data. In addition, it is important to analyze bio-signals in units of year/month/week/day. Therefore, the column-oriented wide column database is easy to retrieve only the information of raw bio-signal data. In addition, this paper designed a bio-signal raw data storage using HBase, which is mainly used in the wide column database.

Raw data on their own have no meaning. They must have more meaningful attributes together such as the time when raw data are measured, whose data are, sampling rates, types of bio-signals, etc., as well as the raw bio-signals such as ECG, respiration, and acceleration data. Table 1 shows an example of the data table that we have designed, using HBase. HBase stores data in a key-value format. Therefore, ‘Row-Key’ specifies the measurement date and ID. The ‘Data’ column is the bio-signal information. The ‘User’ column is the user information that measured the bio-signals. We need information about the types of bio-signals such as electrocardiogram, acceleration, respiration, etc., and the hertz (Hz) which is the sampling frequency of the signal to analyze bio-signals. Moreover, ‘User’ information includes age and gender, since the analysis techniques of bio-signal analysis algorithms vary according to age and gender.

#### 3.1.2. Design of Data Warehouse for Feature Data

Our approach uses HBase to store the vast amount of bio-signal data, depending on the column family. The extracted data (e.g., heart rate variability (HRV)) from raw data (e.g., ECG) are sometimes large as well, and they are stored in the data warehouse. Moreover, we use SQL-On-Hadoop [29] to search and analyze the mined data, which processes the data in a familiar way of interfacing SQL, working with data warehouse-based Hive. Hive uses a similar interface to SQL called HiveQL, and it can be used for statistical analysis.

**Figure 3:**
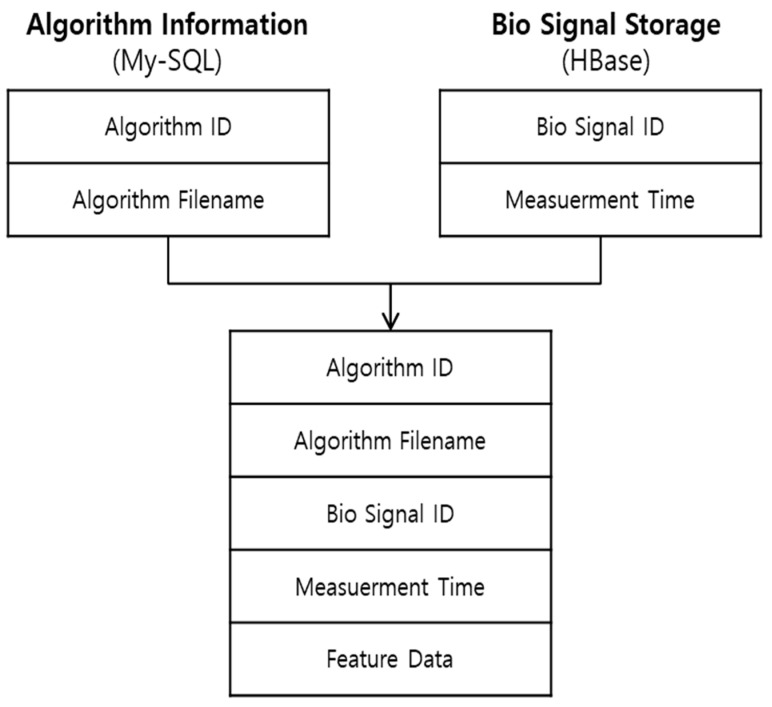
Design of data warehouse structure.

Since Hive is Hadoop-based, its processing speed is much slower if it searches and accesses all the data. Therefore, we use the partitioning method to improve the speed. There is also a need to connect three heterogeneous information types to store the mined and analyzed data in Figure 3. First, the information about the given algorithm to analyze bio-signals in the algorithm information table of MySQL. Second, the information about bio-signals used to analyze in HBase. Third, the extracted feature data in Hive.

Table 2 shows the table structure of Hive. The data are expressed by algorithm ID, algorithm name, the mined data value, and date. In addition, since it is partitioned by bio-signal ID, the acquired year, and month, the search time can be shorter.

### 3.2. Architecture of Bio-Signal Data Mining

Many researchers employ MATLAB to analyze bio-signals, and there is also a recent tendency to use an open-source programming R for big data analysis. Some develop signal processing and mining techniques with MATLAB, others with R programming or with other languages or tools. Data mining techniques are developed in various environments. However, when developers try to use components developed in another language environment than the current development environment, the process of transforming sources in one language to the ones in another is needed, which demands a substantial amount of time for implementing its processor for coding the components working in the development language.

For this reason, it is of great meaning to provide an execution engine enabling to skip the source transforming process, which supports interoperability between different sources. It is particularly important and desirable when one wants to develop systems using vital sign data mining techniques developed in various languages and environments previously. We describe a flow to support interoperability between different (bio-signal) data mining techniques. The execution engine that we designed requires data mining technique specifications where input/output parameters and tool (or language) types are specified to interact with the engine. With such specifications, the execution engine makes the data mining algorithms implemented in different languages work as if they operated in one common environment, resulting in features and other values after executing the sources. 

Among the many development languages, we concentrated on two popular languages, MATLAB and R, which are most frequently used to implement data mining algorithms, as well as Java which is our development language. Both MATLAB and R programming have the advantage in that they support many libraries, GUI, and various ways of expressing the analyzed data and enable the bio-signal analysis with the usage of function-based source files, simultaneously. In our approach, the execution engine helps execute *.m files of MATLAB and *.R of R programming and get feature values from the given bio-signals. 

**Figure 4:**
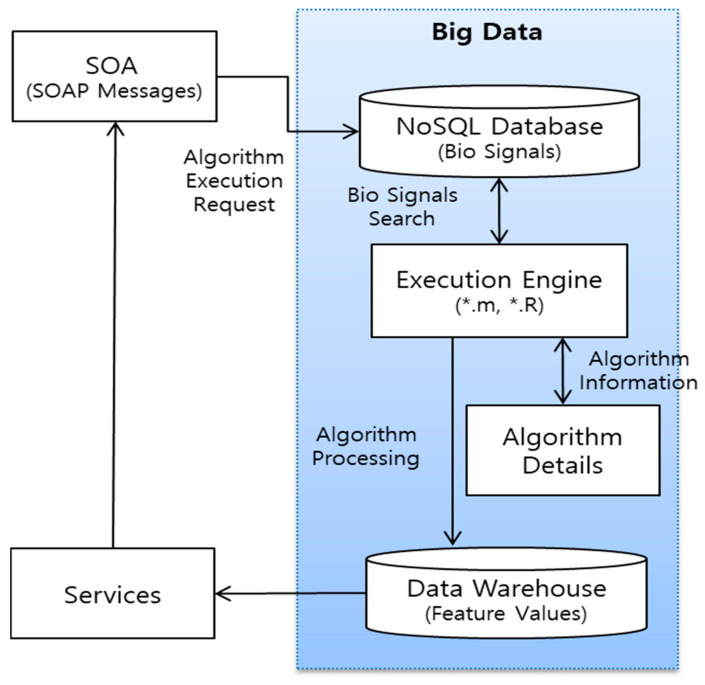
SOA-based bio-signal analysis system architecture.

The proposed architecture is based on a web service model based on a service oriented architecture (SOA) in Figure 4. SOA can be integrated and used without a redundant development of applications that provide various bio-signal services. Therefore, SOA can minimize development costs, and users can easily receive biometric information monitoring services in an integrated environment. In this paper, an SOA-based bio-signal analysis system was developed. The architecture for interoperating among the components consists of the following four processes:

1. Service request for executing the bio-signal analysis algorithm through the simple object access protocol (SOAP) message.
2. Input value, output value, and algorithm explanation for supporting the mining specification of the bio-signal analysis algorithm.
3. Execution engine to run the bio-signal analysis algorithm.
4. Design a data warehouse that stores and classifies the results from the execution engine.

The web-based simple object access protocol (SOAP) message offers the service for executing the bio-signal analysis algorithm. It includes a request to search for raw bio-signals data, a request that uploads the bio-signal algorithm source file developed by using MATLAB or R programming, and a request that extracts the bio-signal feature value by executing the bio-signal analysis algorithm. 

To run the bio-signal analysis algorithm in the system, the parameters and variables of the bio-signal analysis algorithm should be defined. Hence, the algorithm specification describes the information on types of bio-signals to be analyzed (such as ECG, respiration, and acceleration signals), an input value of the bio-signals, result value after the execution of the algorithm, clear explanation of the algorithm, and the developer.

The data warehouse stores the results from the execution engine. The data extracted by applying the bio-signal analysis algorithm may be a great amount in a single column. Therefore, the data is needed to save into the data warehouse for big data analysis.

#### 3.2.1. Data Modeling Specification

To execute data mining modules, an accurate specification of the mining techniques is necessary, since the execution engine works according to the specification in which a type of language (or tool), input/output values, and explanation of the mining algorithms are described. Our study focuses on two well-known languages, MATLAB and R. To understand the execution of source files in these languages, we need to be aware of ‘function’ in MATLAB and R. They help analyze the bio-signal data by providing the functions where various input values are represented, stored, and visualized.

**Figure 5:**
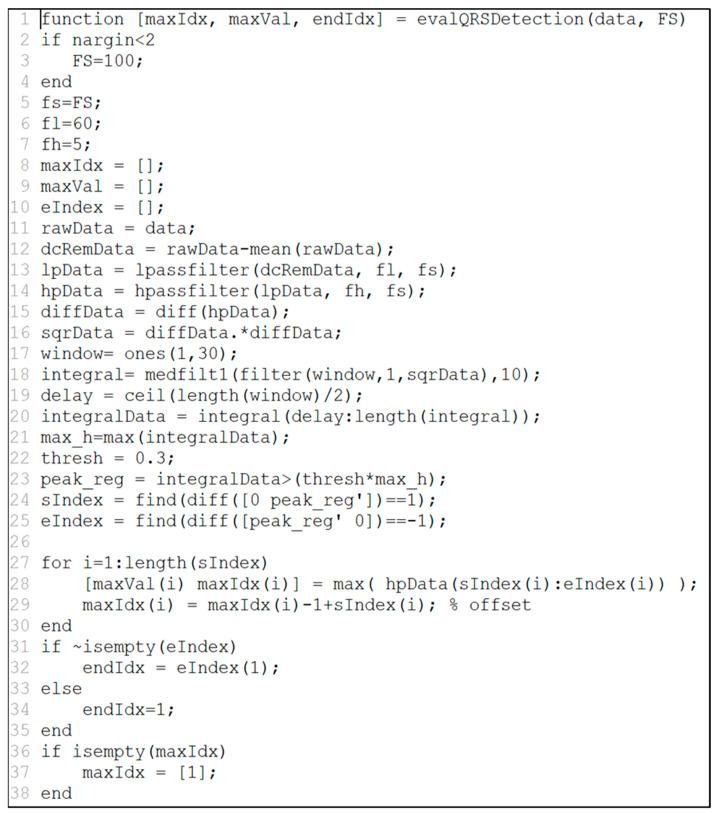
Example of MATLAB source to extract HRV from ECG.

Figure 5 shows an example of MATLAB source code to extract HRV from ECG raw data. In the source, two input values appear such as data (ECG data) and FS (sampling rate). The output values are maxIdx (R-R interval index), maxVal (value of R-R interval), and endIdx (last R-R interval index). MATLAB has a vector data structure so that it can process different types of both input and output variables. For example, it supports several variables to handle ECG such as int, int array, double, and double array.

In general, software specification is a summary of the requirements and functions demanded in the design phase. The mining technique specifications proposed in our work are needed to define and execute the mining techniques in the system. In addition, the mining technique specifications are used to insert, delete, and update input/output parameters, as well as describe the techniques themselves. 

**Figure 6:**
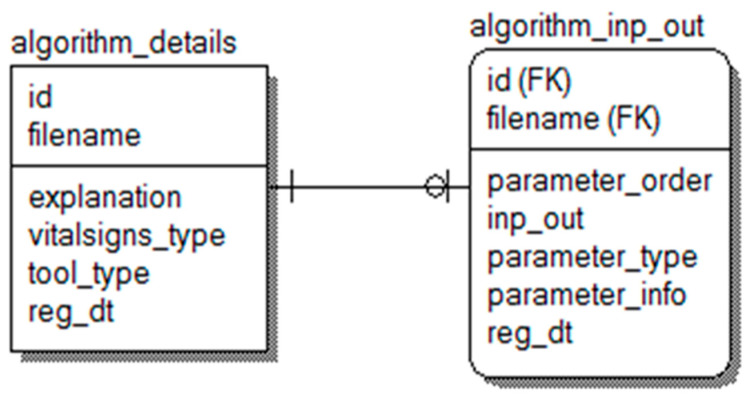
Database modeling for a mining algorithm.

Our approach uses the relational database management system (RDBMS) for specification concerning functions in MATLAB and R programming, so that various input/output values can be defined, inserted, and updated systematically. Figure 6 presents the related database modeling. Table ‘algorithm_details’ represents basic information about a data mining algorithm including ‘id’ (who uploads it), file name, explanation, type of bio-signal, type of analysis tool, and registration date. Here, ‘vitalsign_type’ is a column needed to process various bio-signals, whose values are ECG, respiration, acceleration data, etc. In addition, the tool_type’s values are MATLAB or R to express which bio-signal analysis tool is employed. The table ‘algorithm_inp_out’ is the table where definitions of input/output values are, and one registers what algorithm is applied, the order of parameters (if it is an input value or output value), parameter type, and parameter explanation.

#### 3.2.2. Source File Strategy for the Mining Technique

**Figure 7:**
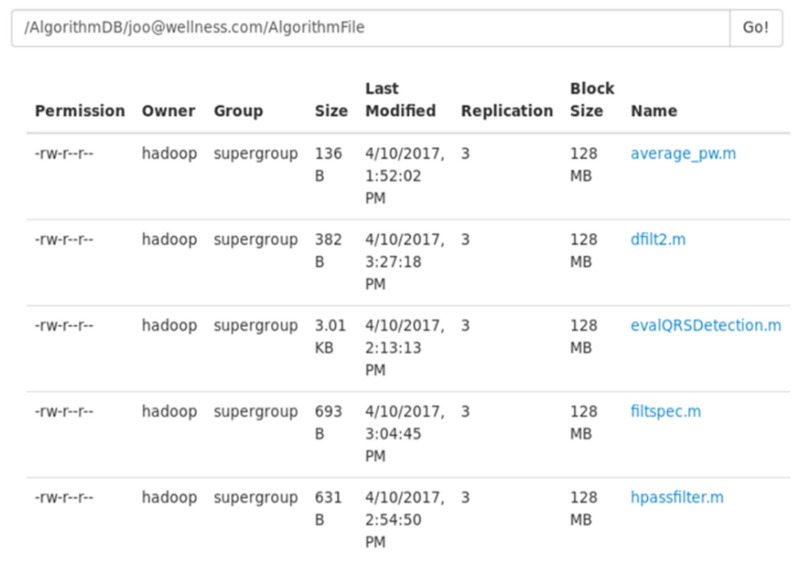
Example of the list of algorithm source files.

Hadoop’s Hadoop distributed file system (HDFS) plays a role in storing mining algorithm source files developed in MATLAB and R, which is suitable for the safe storage of sources. Figure 7 illustrates how source files are stored in Hadoop. It shows that mining algorithm source files are stored under the Hadoop’s folder ‘/AlgorithmDB/’ in Linux to execute algorithm source files (‘joo@wellness.com/AlgorithmFile’). Here, it is necessary to copy the source files in HDFS to Linux or Windows OS by downloading the files in a browser used in HDFS or writing a command. 

#### 3.2.3. Bio-Signal Analysis Algorithm Execution Engine

The bio-signal analysis algorithm source execution technology is a technology that executes a bio-signal analysis algorithm developed by a user, utilizing a bio-signal analysis tool developed in different languages in the system. By omitting the source conversion technology according to the system environment, the environment and interface to execute the source file itself in the system are provided.

**Figure 8:**
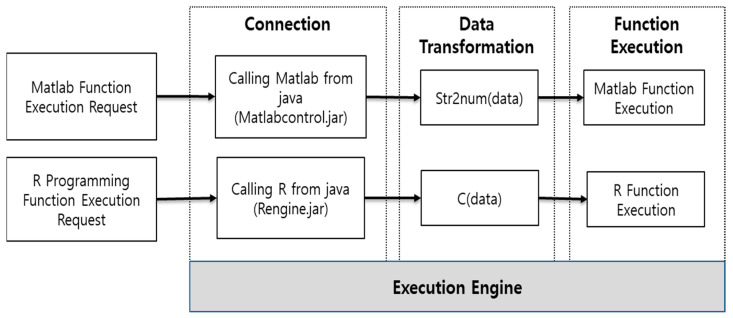
The architecture of functions developed in MATLAB and R programming through Java.

In this paper, we design an architecture that performs the functions developed in MATLAB and R programming using Java, as shown in Figure 8.

The execution technology of the bio-signal analysis algorithm runs the source code developed by MATLAB and R programming in Java. The bio-signal analysis algorithm developed by MATLAB is executed using the MATLAB control library. In addition, the bio-signal analysis algorithm developed by R programming is executed using the Rengine library. Each library is the application programming interface between Java and the development tools.

**Figure 9:**
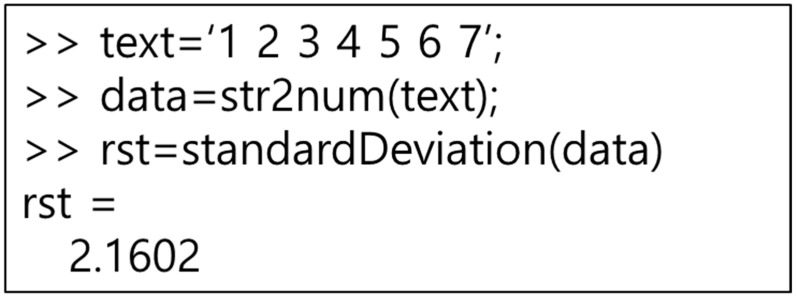
MATLAB function execution command.

To run MATLAB in Java, use the MATLAB control library. The MATLAB control can use MATLAB commands in Java using the eval and f eval methods. The MATLAB control eval and f eval methods can be passed to the MATLAB workspace to execute MATLAB commands. In addition, the result value executed in MATLAB can be returned to Java. The result value returned from MATLAB is returned using the returning eval method of MATLAB controls. This paper accesses MATLAB using Java to execute MATLAB functions. Since the input value to execute the function is inputted through Java, it is inputted in a text format. Therefore, as shown in Figure 9, the input value needs to be converted to a number format through str2 num, a MATLAB method. If the entered value is a text, it can be used as it is. After inputting the input value, execute the MATLAB function, and return the result value to Java for processing.

**Figure 10:**
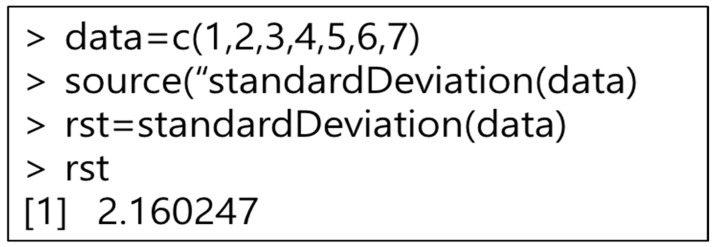
R function execution command.

To execute R programming, we use a method similar to MATLAB. Java uses the Rengine library to access R programming. Rengine can use R programming commands in Java using eval. The R programming eval method can be passed to the R programming workspace to execute R programming instructions. In addition, the result value executed in R programming can be returned to Java. The result value returned from R programming is returned using the eval method. In this paper, we access R programming using Java to execute the R programming function. The input value for executing the function is converted into an R programming input instruction through Java and inputted immediately. Therefore, as shown in Figure 10, since the input value is converted to the c format, there is no need to use a separate conversion function. After entering the input value, execute the R programming function, and return the result value to Java for processing. 

**Figure 11:**
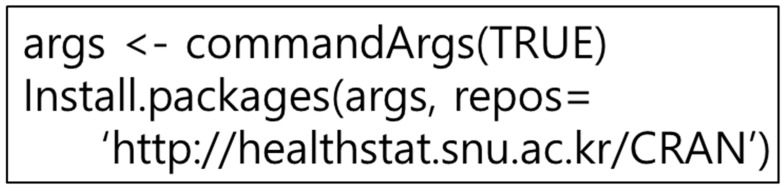
R packages install function.

Since R programming is an open-source type of development tool, various R packages exist. This is important since developers can freely install and use the R packages they need. Therefore, this paper developed a service that can install the R package. However, the R package installation cannot use the Java JRI, since JRI is a Java and R programming interface that allows you to run R in Java applications with a single thread. Here, JRI operates as a single thread, thus installing the R package is physically impossible. Therefore, in this paper, we created the R function in Figure 11 and installed the R package using the Rscirpt of R programming.

## 4. Results

Table 3 measures the execution time of the algorithm source code. It was measured using an electrocardiogram signal among the biological signals. Using data from 60,000 to 150,000 ECG data, one source code was executed a hundred times to obtain an average. The algorithm source code used a lowpass filter developed by MATLAB and R programming to secure universality. As shown in Table 3, the source code is executed directly in R programming and MATLAB runs faster than the source code executed in conjunction with the development tool in Java. The difference in execution speed is to create an input value and pass it to the development tool to execute it in Java. This is due to the fact that it takes time to generate the input value. In addition, it is judged that there is no time difference felt when the bio-signal analysis system is executed.

## 5. Discussion

We can check the results of the feature values through analysis and visualization using the ECG signal. Currently, bio-signals can be measured using various sensors. For example, there are data such as electrocardiogram, brain wave, pulse wave, and acceleration signal. These data have a process of making feature values from raw data and servicing them using feature values. Therefore, the system proposed in this paper can apply various bio-signal data. However, since the algorithm is analyzed using only electrocardiogram data, it is necessary to analyze various bio-signal data such as brain waves and pulse waves. Bio-signal analysis processing can be used in all the versions using a basic analysis module. However, while doing an analysis the version compatibility such as licenses have to be checked, which is a necessary condition. Our framework can be used for all kinds of bio-signals with little customization and also different kinds of analyses can be performed with little or no modification.

In addition, this paper was developed with an emphasis on analyzing feature values through signal processing. However, recently, artificial intelligence technology using train sets and test sets have been widely used. It is necessary to develop a technology that automatically converts the train set and test set to match the bio-signal development language through further research.

## 6. Implementation

**Figure 12:**
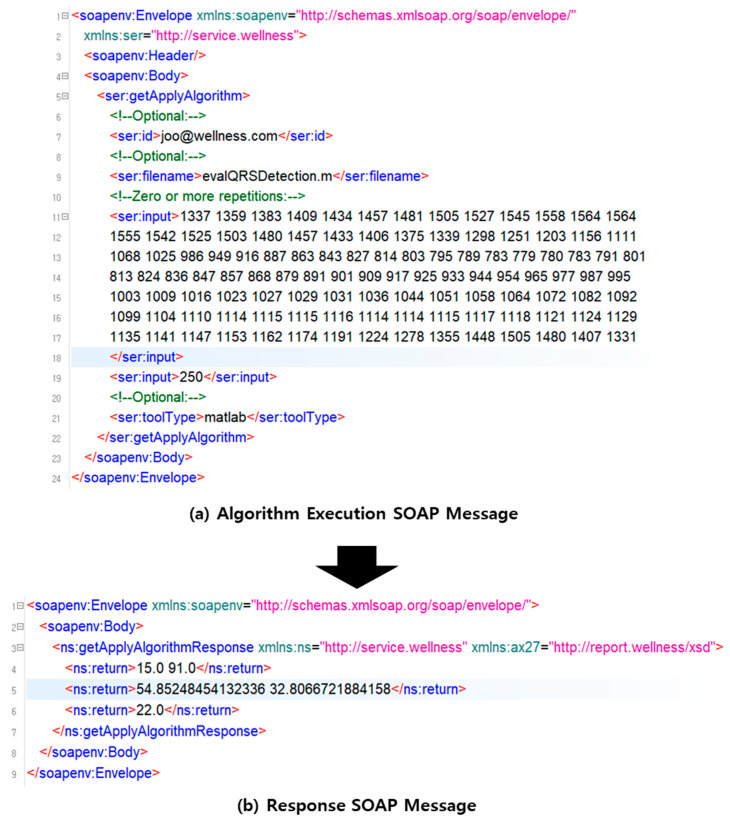
SOAP message of vital sign analysis algorithm execution; (**a**) SOAP message to request the algorithm execution; (**b**) response SOAP message with the algorithm executed.

We have implemented a bio-signal analysis system that can execute SOA-based MATLAB and R programming source codes. The bio-signal analysis system consists of a service that transmits the algorithm developed by MATLAB and R programming to a server and a service that executes the algorithm. In this paper, we have implemented the evalQRSDetection function in Figure 5. Figure 12a is a request SOAP message to execute a function, which includes the function name, input data, and development language. The input values are electrocardiogram data and sampling frequency. The input value can be inputted as the input value of the bio-signal data stored in the bio-signal storage. Moreover, you can input the direct input value. Furthermore, Figure 12b is the response SOAP message. This SOAP message contains the result of executing the function.

**Figure 13:**
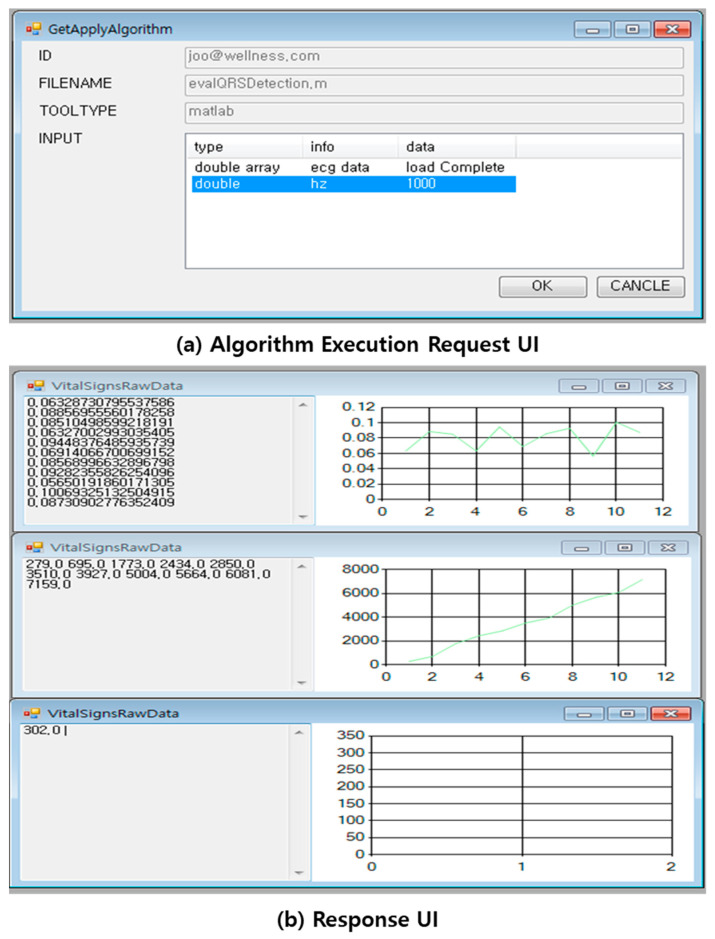
Implementation of vital sign system user interfaces; (**a**) UI for requesting the algorithm execution; (**b**) response UI where the algorithm was executed.

Figure 13 shows the UI for executing the evalQRSDetection function developed in MATLAB and the result UI. Figure 13a is the request UI for executing the evalQRSDetection function. Figure 13b is the response UI showing the result of executing the evalQRSDetection function. The evalQRSDetection function displays three result values. Therefore, we show three UIs for the result values, which show the result data and chart in data format.

## 7. Conclusions

This paper presented an architecture that manages and executes bio-signal analysis algorithms more effectively, with a special focus on interoperability between data mining algorithms developed in heterogeneous environments. While bio-signal analysis components are implemented in different languages such as MATLAB, R, and Java, the proposed platform helps the design teams develop such components and systems as if they were developed in one common language. 

Until now, bio-signal analysts have paid little attention to bio-signals as big data. However, as IoT and wearable technology are rapidly developing, the issue of bio-signals has high potentiality as a research theme for big data processing. Therefore, we need a repository for bio-signals as big data. Secondly, we designed mining algorithm specifications to share algorithms implemented in heterogeneous environments among developers. When design teams implement systems according to such specifications, they are expected to have many benefits, e.g., acquisition of many open algorithms, the overcoming of development restrictions caused by different environments, effective management of bio-signals, and application of the mining algorithms to various and wider environments and languages. In particular, it is expected that healthcare and medical system developers will be able to shorten the system development time using the algorithm execution engine technology. In addition, the real-time execution of various algorithms on the system will be very helpful for system maintenance and management. Thirdly, we developed the execution engine, naturally leading to an advantage that one can execute so many heterogeneous mining techniques with one common system. It also brings about a reduction of the development time and would make people and bio-signal analysts developing in different languages work together. We also hope that communication and competition between algorithms developers are enhanced, and thus higher quality mining technology will be eventually promoted. However, the system presented in this paper has limitations in applying artificial intelligence based on supervised learning. Supervised learning-based artificial intelligence needs to collect and transform a large amount of train set data. The difficulty of transferring large amounts of image files and the study on the application of the conversion technology to train set data are still insufficient. It is judged that such data transmission and conversion technology will be able to find a solution through future research.

Finally, we hope that this research will be fundamental, in which we can go one more step to high-quality, service-oriented research beyond simple signal processing for biodata, by utilizing and developing mining algorithms easily regardless of whatever environments are available.

## Figures and Tables

**Table 1:** HBase table structure.

Row-Key (Measurement Time ID)	Data				User	
	Raw Data	Hz	Type	Overall Time (s)	Age	Gender
2021.03.06.17.420.07_JOO	100 110 112	200	ECG	125	38	Man
2021.03.06.19.01.07_JOO	120 131 110	200	ECG	100	38	Man
2021.03.06.21.28.07_JOO	121 111 120	200	ECG	111	38	Man

**Table 2:** The table structure of Hive.

Partition	Column Name	Data Type	Explanation
X	algorithm_id	String	Algorithm ID
X	algorithm_filename	String	Algorithm file name
X	output	array<String>	Output value
X	day	int	Day
X	hour	int	Hour
X	minute	int	Minute
X	second	int	Second
O	id	String	User ID
O	year	int	Year
O	month	int	Month

**Table 3:** Comparison of execution time using MATLAB, R programming, and Java interface.

ECG Data Amount	R Running Time (s)	JRI Running Time (s)	MATLAB Running Time (s)	MATLAB Control Running Time (s)
60,000	0.01129	0.07554	0.001117402	0.018959
70,000	0.012122	0.07925	0.002032382	0.020659
80,000	0.015542	0.08802	0.002297501	0.021919
90,000	0.014942	0.09725	0.002646774	0.023969
100,000	0.018541	0.12115	0.002932099	0.026389
110,000	0.020813	0.14253	0.003061065	0.029939
120,000	0.022766	0.14519	0.003414657	0.032159
130,000	0.023421	0.15199	0.003779691	0.034149
140,000	0.023759	0.16123	0.004370618	0.036269
150,000	0.025931	0.17423	0.004643073	0.038039

## Data Availability

The data are available on request.
